# *Cinnamomum verum* and *Coriandrum sativum* secondary metabolites exhibit neuroprotective potential: insights from in vitro, ex vivo and computational models

**DOI:** 10.1007/s40203-025-00412-7

**Published:** 2025-08-27

**Authors:** Ayodeji O. Falade, Megan D. Akingbasote, Kayode E. Adewole, Odunayo M. Agunloye, Ahmed A. Ishola, Aimen Aljoundi, Ghazi Elamin, Kolawole A. Olofinsan

**Affiliations:** 1 Biotechnology, Computational Biochemistry and Phytomedicine Research Group, Department of Biochemistry, University of Medical Sciences, Ondo, Ondo State Nigeria; 2 Functional Foods, Nutraceuticals and Phytomedicine Unit, Department of Biochemistry, Federal University of Technology, Akure, Ondo State Nigeria; 3 Central Research Laboratories Limited, 132B, University Road, Ilorin, Kwara State Nigeria; 4 Department of Medicinal Chemistry, College of Pharmacy, Attahadi University, Tripoli, V567-7M8 Libya; 5 Department of Medicinal Chemistry, Faculty of Pharmacy, University of Tripoli, Tripoli, Libya; 6 Department of Anaesthesiology & Critical Care, Nelson R Mandela School of Medicine, University of KwaZulu-Natal, Durban, South Africa; 7 Department of Pharmacology, University of the Free State, Bloemfontein, 9300 South Africa

**Keywords:** Alzheimer’s disease, Antioxidants, Cholinesterase inhibitors, Molecular dynamics, Neurodegenerative disorders

## Abstract

Cinnamon and coriander plants are sources of popular spice products in different cuisines prepared by many people worldwide. The inhibitory effect of cinnamon bark and coriander seeds aqueous extracts on butyrylcholinesterase (BChE) and acetylcholinesterase (AChE) activities, and their antioxidant properties were investigated using in vitro, molecular docking and molecular dynamics (MD) computational models. Standard experimental methods were employed to determine the plant extracts’ antioxidant and enzyme-inhibitory capacities. Subsequently, chemically annotated metabolites from the extracts were subjected to MD with the enzymes before the ligand–protein complexes of two compounds with the highest docking scores were subjected to MD at 300 ns. Phytochemical profiling of extracts from the plants via Gas Chromatography–Mass Spectrometry analysis revealed the presence of polyphenols, flavonoids, terpenes and their derivatives. The extracts from the plant inhibited the investigated cholinergic proteins, although no significant differences (*p* > 0.05) were observed in the IC_50_ values for their activities. While both extracts demonstrated good antioxidative activities, the cinnamon extract exhibited better radical scavenging and Fe-chelation abilities, while the coriander extract exhibited superior ferric-reducing properties. Amongst the chemical compounds identified from the plants' extract, chlorogenic acid with − 31.87 kcal/mol & − 39.29 kcal/mol and rutin with − 48.27 kcal/mol & − 54.37 kcal/mol MM/GBSA computed scores exhibited more negative binding affinity, thus showed potential to be the dominant inhibitors of the AChE and BChE enzymes, respectively. The 300 ns MD results revealed that the proteins' structure was stable after binding of the spice phytoconstituents. Further activity-guided isolation experiment is required to determine the spices as viable sources of these neuroprotective polyphenols.

## Introduction

Neurodegenerative disorders are diseases that cause impairment in normal brain functions. This pathophysiology, among others, includes Alzheimer's disease (AD), Parkinson's and Huntington’s diseases. However, AD seems the most common (Kaur et al. 2017). AD is a multifaceted neuro disorder common in older people (> 65 years) and is responsible for about 50–60% of dementia cases (Kaur et al. 2017). It devastates neuro-signalling while adversely affecting nerve communication, memory and cognition (Soelter et al. 2024).

Even though the actual cause of AD is unknown, redox imbalance, inflammation, degradation, and protein aggregation have been implicated in its pathogenesis (Azargoonjahromi 2024; Soelter et al. 2024). Furthermore, AD is associated with the shortage of acetylcholine, a key molecule that functions in brain nerve impulse transmission (Adedayo et al. 2021). The decrease in this neurotransmitter, which happens at the brain synaptic gaps of AD patients, emanates from cholinesterases continuous breakdown of acetylcholine. Thus, BChE and AChE cholinesterases are promising targets for developing therapeutics in managing AD. Interestingly, this strategy has birthed different inhibitors of cholinergic enzymes (ChEIs) like galantamine, rivastigmine, donepezil, and tacrine, which are now being used as drugs for treating AD patients (Ehab et al. 2019). Although these aforementioned drugs are effective, they are characterized by some adverse effects (Ehab et al. 2019). Hence, the significance of searching for more effective ChEIs from natural sources with little or no adverse effects.

Several scientific studies have highlighted the importance of medicinal plants as sources of pharmacological active molecules for mitigating the dysregulated biochemical parameters associated with neurodegenerative disorders (El-Nashar et al. 2022, 2024). More specifically, some others have evidenced culinary plant spices as natural sources of biological compounds with a variety of other health benefits, including antioxidant properties (Embuscado 2019), anti-diabetic potential (Mnge et al. 2025), anti-carcinogenic, chemoprotective (Kothawade et al. 2024) and nephroprotective activities (Wang et al. 2025). Besides the culinary uses of spices, they are also utilized in ingenious medicine to manage different diseases. Accordingly, the incidence of some neurological disorders has been inversely correlated with regular consumption of spices in the Asian subcontinent (Norouzkhani et al. 2022).

Cinnamon and coriander are commonly consumed spices globally and have exhibited promising medicinal properties. These two spices have been used traditionally in different countries to improve memory and cognitive functions (Mathew and Subramanian 2014). However, there is a dearth of information on the mechanisms that underpin their usage in the therapy of neurodegenerative disorders. Therefore, this study sought to determine the cholinesterase inhibitory and antioxidative activities of aqueous extracts prepared from commercially available cinnamon powder and coriander seeds using in vitro and computational analysis methods.

## Materials and methods

### Collection of spice plant samples and preparation

Commercially available cinnamon (*Cinnamomum verum*) bark and coriander (*Coriandrum sativum*) seeds were purchased from a culinary spice store in Ondo market, Nigeria. The food samples were authentic at the Centre for Research and Development, Federal University of Technology, Akure, before specimens of each plant product, with voucher numbers 0297 (cinnamon) and 0298 (coriander), were deposited at the institution's herbarium. After authentication, the plant samples were air-dried and powdered. Subsequently, 0.5 g of each sample was agitated on a HY 4-speed governing multipurpose oscillator (Tech Instruments, UK) in 0.5 L bottles containing 0.25 L distilled water (0.25 L) for 24 h. After running the mixture through a sieving mesh, further filtration was done with a Whatman filter paper. The resulting extracts employed for the in vitro assays were obtained from these filtrates and air-dried after concentration *in vacuo*.

### Animal experiment and tissue homogenate preparation

Male rats of the Wistar strain (n = 6) weighing 220 ± 10.9 g were obtained for the animal house unit of the Department of Biochemistry, Federal University of Technology, Akure. The animals were fasted for the entire night before being euthanized, and their brain tissues were subsequently removed. After that, the removed tissues were homogenized in 100 mM, pH 6.9 phosphate buffer before centrifuging (4 °C) for 15 min at 12,000 × *g*. The supernatants recovered were used for the cholinesterase enzyme inhibition experiment. The protocol for this study was approved by the Health Research Ethics Committee of the University of Medical Sciences, Ondo, Nigeria (EAC14/2021) while complying with the National Institutes of Health guidelines for the care, handling and use of laboratory animals.

### Enzyme assay

The BChE and AChE inhibitory capacity of donepezil, a standard drug with coriander seeds and Ceylon cinnamon bark aqueous extracts, were evaluated using the previous method of Oboh et al. (2017). 50–150 μL varying concentrations of the extract samples were incubated (25⁰C for 20 min) with 200 µL of enzyme solution (rat brain homogenate) and 3.3 mM 5,5'-dithio-bis(2-nitrobenzoic) acid (100 µL). 50 μM of 100 µL of butyrylthiocholine iodide and acetylthiocholine iodide substrate solutions were added to the reaction mixture containing BChE and AChE enzymes, respectively. The change in absorbance was read at 412 nm for 180 s in a spectrophotometer and plotted against time to estimate the cholinergic enzyme activity. While a mixture without the extracts served as the control, the % enzyme activity inhibition is determined with the expression below:

$$ \% Inhibition = \frac{{\left( {\frac{\Delta Acontrol}{{\Delta t}}} \right) - \left( {\frac{\Delta Asample}{{\Delta t}}} \right)}}{\Delta Acontrol/\Delta t} X 100 $$

where ΔA_control_ = change in absorbance @ 412 nm of control. ΔA_sample_ = change in absorbance @ 412 nm of the sample. Δt = change in time.

### In vitro *antioxidant assays*

#### Hydroxyl (OH) radical scavenging ability

The extracts’ capacities in donating electrons to hydroxyl radicals compared to the Vitamin C standard compound were evaluated using the Halliwell and Gutteridge (1981) method, detailed in the report by Oboh et al. (2017).

#### 2,2′-azino-bis (3-ethylbenzothiazoline-6-sulfonic acid) (ABTS) scavenging ability

A solution containing radical species of this compound was produced after a reaction mixture containing K_2_S_2_O_8_ (2.45 mM) and 7 mM ABTS solution was incubated for 16 h in the dark, followed by its absorbance (734 nm) adjusted to 0.700 with ethanol. The extracts’ abilities to scavenge ABTS were compared with Trolox using Re et al. (1999). About 200 μL of the extract samples were added to 0.2 mL of ABTS solution before reading the absorbance at 734 nm after 15 min. Subsequently, the % ABTS radical scavenging ability was determined.

#### Ferric (Fe^3+^) reducing antioxidant property (FRAP)

The reducing power of the extracts was evaluated using the Oyaizu (1986) method, as documented previously by Oboh et al. (2017). Then, the ability of the samples to reduce Fe3 + ions was estimated as mg ascorbic acid equivalent (AAE) per gram dry sample.

#### Ferrous (Fe^2+^)-chelating capacity

The capacity of the extracts to chelate Fe (II) was determined at 510 nm using the Puntel et al. (2005) method described previously by Oboh et al. (2017).

#### Determination of total phenol and flavonoid contents

The total phytochemicals present in the extracts were determined by quantifying the total flavonoid and polyphenol concentration of the extracts using the respective methods by Meda et al. (2005) and Singleton et al. (1999).

#### Characterization of extracts’ phytoconstituent

The possible bioactive components of the cinnamon and coriander extracts were analyzed via gas chromatography–mass spectrometric (GC–MS) according to Chipiti et al. (2015) outlined protocol. The extracts to be analyzed were initially derivatized via silylation as described by Proestos and Komaitis (2013). Then, analysis was carried out using a Varian 3800 gas chromatograph (Agilent Technology, USA) equipped with an Agilent fused silica capillary CP-Sil 5 CB column (30 m × 0.25 mm i.d.) connected to a Varian 4000 mass spectrometer operating in the EI mode (70 eV; m/z 30–800 amu; source temperature 230 °C and a quadruple temperature 150 °C). Nitrogen gas was employed as a carrier gas for the derivatized samples (1µL) injected in 10:1 split mode for a total run period of 40–55 min at a flow rate of 0.8 mL/min. Phytochemical components of the samples were identified by cross-referencing the analysis-generated data with those found in the National Institute of Standard and Technology library database.

### In silico study

#### Preparation of cholinergic protein targets

The chemical structures of butyrylcholinesterase (PDB ID: 4B0P) described by Gerlits et al. (2019) and acetylcholinesterase (PDB ID: 6O4W) reported by Wandhammer et al. (2013) were retrieved from the RSCB Protein Data Bank (Berman et al. 2002) at http://www.rcsb.org. UCSF Chimaera was used to eliminate all cofactors, non-standard residues such as Cl^−^, Na^+^, and water molecules surrounding the cholinesterase protein structures (Pettersen et al. 2004). In order to process the downloaded structures, their attached ligand and water molecules were removed before the Autodock program (v4.2) of Scripps Research Institute was employed to add hydrogen atoms. The search grid was extended beyond the subject proteins, and the characteristics of the atomic solution were" resolved. Gasteiger-type polar hydrogen charges were assigned; the non-polar ones were merged with the carbons, while the internal degrees of freedom and torsion were generated before the final minimized structure was saved in pdbqt format.

#### Preparation of ligand molecules

The chemical structure of donepezil, galantamine and the phytoconstituents identified in the spices were retrieved at www.pubchem.ncbi.nlm.nih.gov. Then, Open Babel software (O'Boyle et al. 2011) was employed to transform their downloaded SDF file format to mol2. Then, Avogadro software was used to optimize the geometry of the ligand molecules by zeroing their torsion and internal degrees of freedom after α-carbon identification. Thereafter, each compound's modified structure was saved in pdbqt format for docking into the binding pockets of AChE and BChE (Hanwell et al. 2012).

#### Molecular docking with cholinergic protein targets

This stage of the in silico analysis involving the prepared compounds with the BChE and AChE proteins was done using Trott and Olson (2010) Vina Graphic User Interface programme. The search volume dimension for BChE docking was set at X: 31.98, Z: 25.95, Y: 18.61 centre values with 64.67 × 82.29 × 65.76 grid box values, whereas for AChE, the centre parameters were X: 91.89, Z: -15.94, Y: 84.37, but with 80.71 × 59.62 × 78.89 grid box measurement. Structural poses containing clusters with the lowest binding energy were selected during the Vina program runs based on Root Mean Square Deviation (RMSD) assigned energy values. Then, the molecular interaction between the ligand and the BChE and AChE protein active site amino residue was processed with Discovery Studio Visualizer, 2020.

#### Molecular dynamics simulation

After the molecular docking analysis, the protein–ligand complex of the two compounds (chlorogenic acid and rutin) from the plant extract with the lowest binding energy was subjected to molecular dynamics simulation. Subsequently, a system consisting of each compound bound to BChE and AChE underwent a 300 ns MD simulation with the AMBER18 CPU and GPU packages (Aljoundi et al. 2020, 2021), PMEMD engine. An atomic partial charge was generated for seven ligands using the AMBER FF14SB of the ANTECHAMBER program (Wang et al. 2004). The Leap module of Amber 20 enabled the addition of hydrogen atoms, sodium (Na^+^), or chloride (Cl^−^) counter ions to the systems for neutralization. The systems were initially minimized for 2500 steps with 500 kcal/mol Å^2^ restraint potential, and a whole minimization step of 5000 steps was further run without restraint using the conjugate algorithm. Thereafter, the system underwent gradual heating from 0 to 300 K for 50 ps by means of a Langevin thermostat in a canonical assemblage (NVT) (Grest and Kremer 1986; Metwally et al. 2023). Following heating, equilibration was undertaken at a temperature of 300 K, excluding all restraints, and a maintained atmospheric pressure of 1 bar by utilizing the Berendsen barostat until all systems had reached equilibration (Berendsen et al. 1984). The complexes then underwent a 300 ns MD simulation with the SHAKE algorithm being employed to constrain all hydrogen atoms (Elamin et al. 2022; Ryckaert et al. 1977). The generated trajectories of each system were analyzed using the PTRAJ and CPPTRAJ modules (Roe and Cheatham 2013). The generated data and subsequent complexes were visualized using Microcal Origin analytical software (Aljoundi et al. 2020; Roe and Cheatham 2013).

### Molecular dynamics simulation post-analysis

The bound complexes were saved after every 1 ps, and their resultant trajectories were analyzed using the AMBER 20 integrated CPPTRAJ module. Post-analysis for the systems MD simulation included protein, stability (RMSD), flexibility (RMSF) and solvent-accessible surface area (SASA). Binding free energy is computed by employing the Molecular Mechanics/Generalized Born Surface Area (MM/GBSA) technique.

### Binding free energy calculations

To determine an estimate of the binding free energy of the BChE and AChE bound to two inhibitors, a method known as the Molecular Mechanics/Generalized Born Surface Area (MM/GBSA) approach has been implemented. The binding free energy represented by ΔG_bind_ is a reliable tool that is extensively used to measure the energies that contribute to the binding of the protein and ligand to form a complex, and is calculated using the following equation:

$$ \Delta {\text{G}}_{{{\text{bind}}}} = {\text{ G}}_{{{\text{complex}}}} {-}{\text{ G}}_{{{\text{protein}}}} {-}{\text{ G}}_{{{\text{inhibitor}}}} $$

$$ \Delta {\text{G}}_{{{\text{bind}}}} = {\text{ E}}_{{{\text{gas}}}} + {\text{ G}}_{{{\text{sol}}}} {-}{\text{ TS}} $$

The ΔG_bind_ is the sum of the gas and solvent energy minus entropy (TS).

$$ {\text{E}}_{{{\text{gas}}}} = {\text{ E}}_{{{\text{int}}}} + {\text{ E}}_{{{\text{vdw}}}} + {\text{ E}}_{{{\text{ele}}}} $$

where E_gas_ is representative of the addition of the internal energy terms of the AMBER force fields, which includes E_int,_ which represents angles, torsions, and bonds, E_vdw,_ which represents covalent van der Waals energy and E_elec,_ which represents the electrostatic energy components that are non-bonded.

The following equation is representative of the solvent energy calculation:

$$ {\text{G}}_{{{\text{sol}}}} = {\text{ G}}_{{{\text{GB}}}} + {\text{ G}}_{{{\text{SA}}}} $$

$$ {\text{G}}_{{{\text{SA}}}} = \gamma {\text{SASA}} $$

G_GB_ is representative of the polar solvation impact, and G_SA_ is representative of the non-polar solvation impact, which is calculated using the solvent-accessible surface area (SASA). This is attained by using a water probe radius of 1.4 A°. A surface tension constant (c) was represented by a measure of 0.0072 kcal/mol, and ‘b’ to 0 kcal/mol. The per-residue energy decomposition evaluation was also conducted to estimate the energy contribution of individual residues at the catalytic site, contributing to the compounds’ stability and affinity. This provides much atomistic insight into the inhibition demonstrated by the inhibitors, given that a high residual energy contribution is indicative of critical residues.

### Data analysis

Generated experimental data were subjected to non-parametric unpaired t-tests using GraphPad Prism version 5.0. at 95% confidence intervals. This software was also employed to calculate FRAP assay EC_50_ (sample concentration that can exhibit 50% of FRAP capacity) and IC_50_ values (sample concentration causing 50% inhibition) for the other assays. The difference between means was accepted as significant at *p* < 0.05.

## Results

### Anti-cholinesterase activities of cinnamon bark and coriander seed extracts

**Fig. 1:**
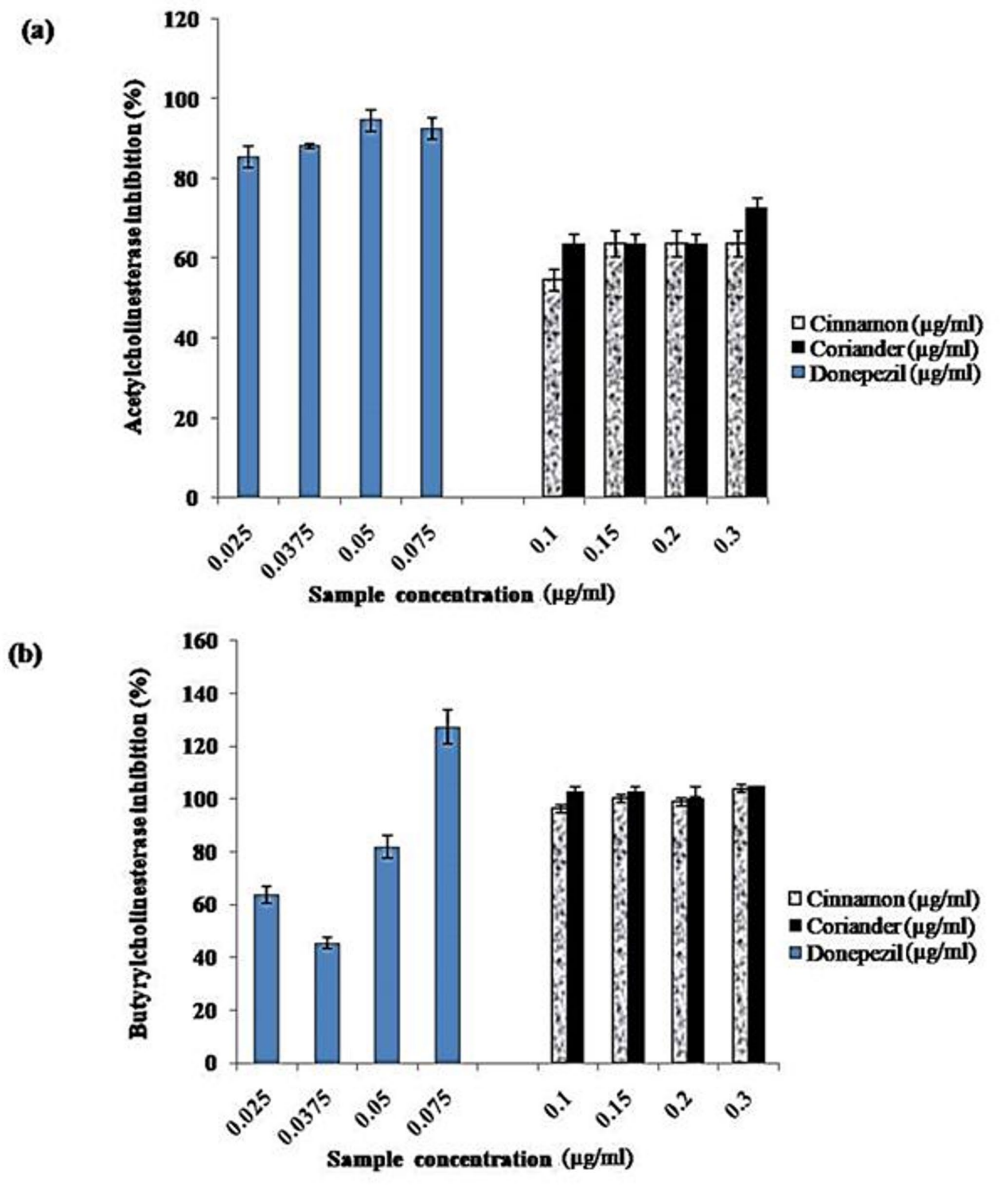
Effect of cinnamon bark and coriander seed extracts on **a** acetylcholinesterase and **b** butyrylcholinesterase enzymes

The results from this study, as presented in Fig. 1a–b, indicate that aqueous extracts from cinnamon powder and coriander seeds demonstrated remarkable inhibitory activities against the cholinesterases. While the spice extracts exhibited over 60% AChE inhibition at 0.15–0.30 μg/mL, they almost completely inhibited BChE at the same experimental concentrations. Table 1 showed no significant differences (*p* > 0.05) in the BChE inhibitory IC_50_ values of the extracts. Despite the BChE overall strong inhibitory activities of the extracts, their higher IC_50_ values with AChE in Table 1 suggest their lesser efficacy than donepezil, representing a standard cholinesterase inhibitor.

**Table 1:** IC_50_ and the EC_50_ values of cinnamon bark and coriander seed extracts' biological activities

	AChE	BChE	OH scavenging	FRAP	Fe^2+^-chelating
Cinnamon (µg/mL)	0.091 ± 0.05^a^	0.024 ± 0.36^a^	92.09 ± 39.21^a^	14.82 ± 1.73^a^	20.60 ± 1.85^a^
Coriander (µg/mL)	0.073 ± 0. 04^a^	0.023 ± 0.56^a^	210.90 ± 79.60^b^	11.52 ± 1.57^b^	24.59 ± 2.15^b^
Donepezil (µg/mL)	0.008 ± 0.03^b^	0.01 ± 0.04^b^	–	–	–
Vitamin C (µg/mL)	–	–	0.05 ± 0.04^c^	–	–

Column data with different letter a–b subscripts are significantly different (*p* > 0.05). EC_50_ of FRAP indicates the extract concentration that exhibits 50% of FRAP capacity. IC_50_ values: extract concentration that caused 50% inhibition

### Antioxidant properties of cinnamon bark and coriander seed extracts

**Fig. 2:**
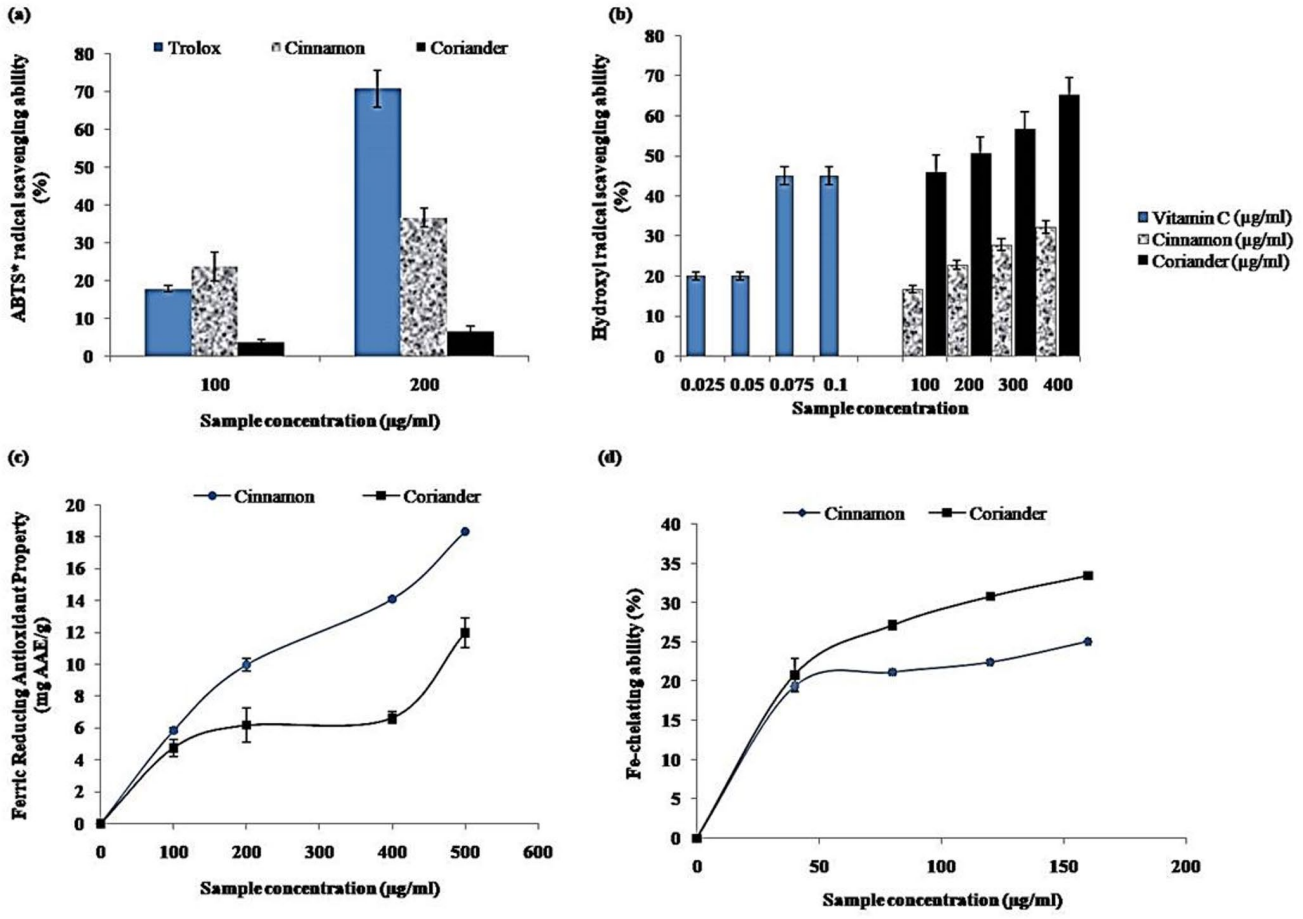
**a** ABTS radical scavenging ability, **b** Hydroxyl radical scavenging ability, **c** Ferric reducing antioxidant property and **d** Fe-chelating ability antioxidant properties cinnamon bark and coriander seed aqueous extracts

Data in Fig. 2a–b and some IC_50_ values in Table 1 also showed the abilities of these plant extracts to scavenge OH and ABTS radical species. It could be observed that both extracts exhibited ABTS and OH radicals scavenging capabilities in a concentration-dependent manner (Fig. 2a–b). However, cinnamon powder extract displayed a better ABTS radical scavenging ability (100 µg/mL: 23.74% and 200 µg/mL: 36.67%) compared to coriander seeds extract, with 3.82 and 6.75% inhibition at 100 and 200 µg/mL, respectively. Still, both extracts' ABTS radical scavenging capacities were significantly lower than the standard: Trolox at higher concentration (70.73%). Similarly, cinnamon extract had a stronger ability to scavenge OH radicals, as reflected in the IC_50_ value (cinnamon: 92.09 µg/mL; coriander: 210.90 µg/mL). Nevertheless, vitamin C exhibited a more potent OH radical scavenging property (IC_50_: 0.05 µg/mL) than both extracts. More so, the extracts reduced Fe^3+^ to Fe^2+^, which was, in turn, chelated by the extracts (Fig. 2c–d). The extracts’ Fe^2+^-chelating and FRAP capacities increased with the sample concentrations (Fig. 2c–d). Judging from the respective EC_50_/IC_50_ values of the extracts (Table 1), coriander seeds extract exhibited a better FRAP, while cinnamon powder extract had a higher Fe^2+^-chelating ability (Table 1).

### Phytoconstituents of cinnamon bark and coriander seed extracts

**Fig. 3:**
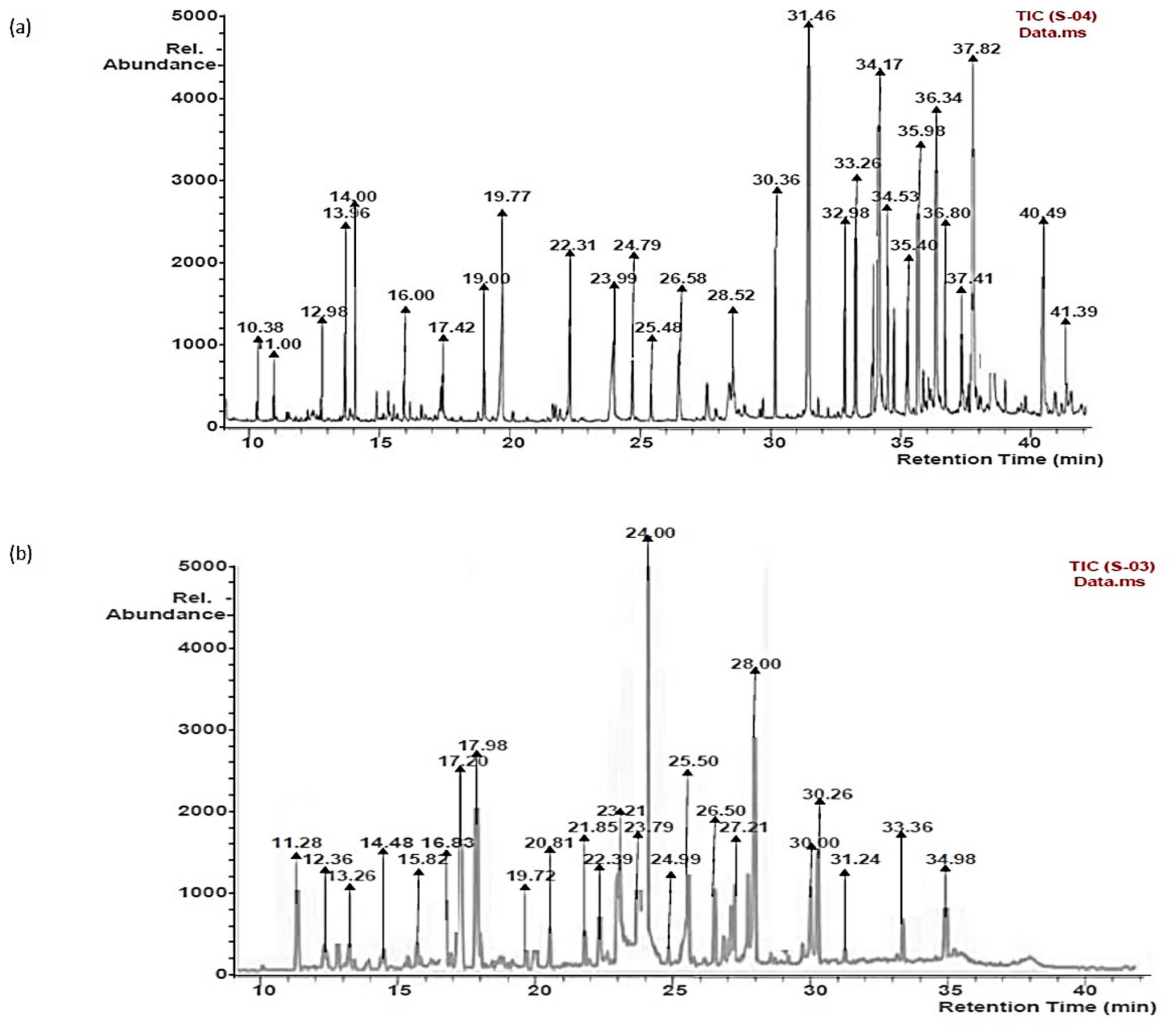
The Total Ion Chromatograms of **a** cinnamon bark and **b** coriander seed extracts chemical constituent analysis

**Fig. 4:**
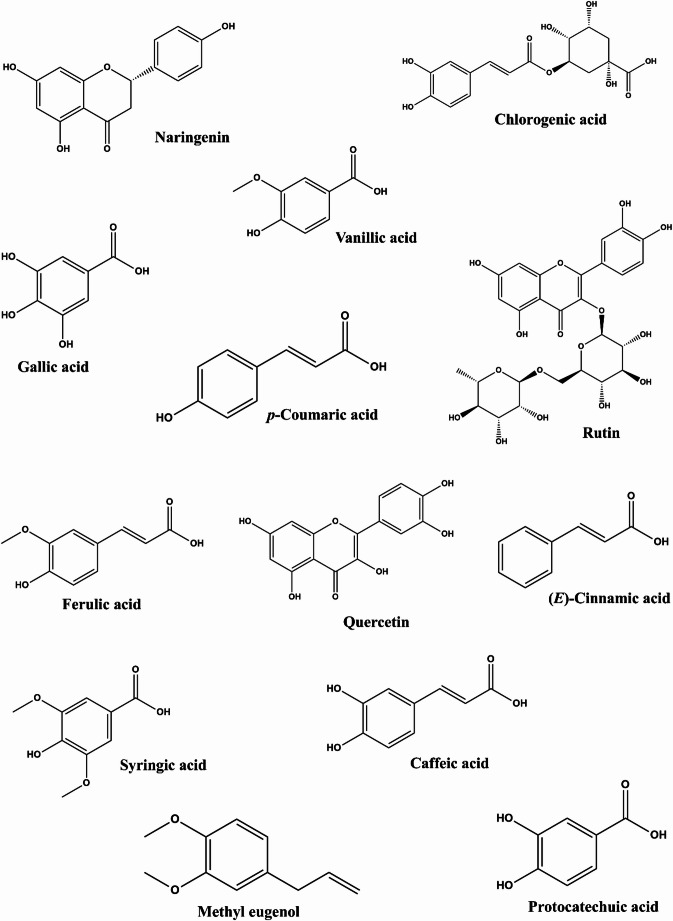
2D structural images of various chemical compounds found in the investigated cinnamon bark and coriander seed extracts

To quantify the bioactive constituents in the extracts, the total flavonoid and phenol concentrations were determined, as presented in Table 2. The extract from cinnamon contained higher total phenols (9.92 ± 0.16 mg GAE/g) than the coriander seeds extract (4.80 ± 0.08 mg GAE/g). However, coriander seed extract contained higher total flavonoid (2.66 ± 0.05 mg QE/g) than cinnamon extract (0.31 ± 0.00 mg QE/g). Furthermore, characterization of the extracts using GC–MS revealed various classes of bioactive phytochemical compounds, including polyphenols, flavonoids, terpenes and their derivatives in the cinnamon powder and the coriander seeds extracts as displayed in Table 3, with their respective chromatograms in Fig. 3. According to our search through various scientific databases and to the best of our knowledge eucalyptol has not been reported in cinnamon bark while nerolidol, coumarin, 2,3-dihydroxybenzoic acid and p-hydroxybenzaldehyde phytochemicals have not been documented in coriander seeds plant material. Previous reports in line with our analysis findings relating to the presence of the compounds in the plant products are indicated in Table 3. Moreover, Fig. 4 presents the structural diversity of compounds found in the studied cinnamon and coriander extracts.

**Table 2:** Cinnamon bark and coriander seed aqueous extracts phytochemical concentration

	Total flavonoid (mg QE/g)	Total phenol (mg GAE/g)
Cinnamon	0.31 ± 0.00^a^	9.92 ± 0.16^a^
Coriander	2.66 ± 0.05^b^	4.80 ± 0.08^b^

Column data with different letter a–b subscripts are significantly different (*p* > 0.05)

**Table 3:** Phytochemical constituents of cinnamon bark and coriander seed extracts from this study compared with previous reports

Compounds	molecular weight	Chemical formular	Coriander seed	Peak area (%)	Cinnamon bark	**Peak Area (%)**
Linalool	154	C_10_H_18_O	Caputo et al. (2021)	19.03	–	ND
Methyl eugenol	178	C_11_H_14_O_2_	López et al. (2008)	11.47	Wijesekera and Chichester (1978)	1.67
Protocatechuic acid	154	C_7_H_6_O_4_	Scandar et al. (2023)	7.65	Pramote et al. (2012)	1.42
Carvone	150	C_10_H_14_O	Samojlik et al. (2010)	6.02	–	ND
(*E*)-isoeugenol	164	C_10_H_12_O_2_	–	5.06	Wijesekera and Chichester (1978)	0.61
Caffeic acid	180	C_9_H_8_O_4_	Scandar et al. (2023)	4.59	Pramote et al. (2012)	9.17
Syringic acid	198	C_9_H_10_O_5_	Sriti et al. (2012)	3.82	Leela (2008)	2.92
Ferulic acid	194	C_10_H_10_O_4_	Nambiar et al. (2010)	3.81	Pramote et al. (2012)	7.50
*p*-coumaric acid	164	C_9_H_8_O_3_	Scandar et al. (2023)	3.54	Pramote et al. (2012)	2.50
(*E*)-cinnamic acid	148	C_9_H_8_O_2_	Scandar et al. (2023)	2.95	Wijesekera and Chichester (1978)	1.25
Rutin	664	C_27_H_36_O_19_	Sriti et al. (2012)	2.41	Gunawardena et al. (2014)	1.02
Vanillic acid	168	C_8_H_8_O_4_	Nambiar et al. (2010)	2.33	Pramote et al. (2012)	4.79
Chlorogenic acid	354	C_16_H_18_O_9_	Scandar et al. (2023)	1.91	Selim et al. (2024)	1.58
Eucalyptol	154	C_10_H_18_O	Micić et al. (2019)	1.51	–	ND
*p*-Hydroxybenzaldehyde	122	C_7_H_6_O_4_	–	1.50	Dvorackova et al. (2015)	0.86
2,3-dihydroxybenzoic acid	154	C_7_H_6_O_4_	–	1.49	–	ND
Gallic acid	170	C_7_H_6_O_5_	Sriti et al. (2012)	1.15	Verma et al. (2021)	2.92
Quercetin	302	C_15_H_10_O_7_	Nambiar et al. (2010)	0.80	Gunawardena et al. (2014)	7.09
Cinnamaldehyde	132	C_9_H_8_O	–	ND	Wijesekera and Chichester (1978)	6.07
Scopoletin	192	C_10_H_8_O_4_	–	ND	Oganesyan et al. (2007)	4.17
Nerolidol	222	C_15_H_26_O	–	ND	Leela (2008)	3.79
Naringenin	272	C_15_H_12_O_5_	–	ND	Farag et al. (2022)	2.51
(*Z*)-2-methoxycinnamaldehyde	162	C_10_H_10_O_2_	–	ND	Wijesekera and Chichester (1978)	2.03
4-hydroxy-2-methoxycinnamaldehyde	178	C_10_H_10_O_3_	–	ND	–	1.27
Coumarin	146	C_9_H_6_O_2_	–	ND	Wijesekera and Chichester (1978)	0.83

*ND* Not determined

### Molecular docking of detected bioactive compounds with AChE and BChE

In Table 4, three phenolic compounds displayed remarkable binding affinities during the molecular docking simulation with AChE. Chlorogenic acid, rutin and naringenin had more negative binding affinities (− 12.4 kcal/mol, − 11.7 kcal/mol and − 10.9 kcal/mol, respectively) for AChE compared to galantamine (− 10.8 kcal/mol), a standard cholinesterase inhibitor. Similarly, the three aforementioned compounds and quercetin were outstanding in their binding affinities for BChE (Table 4). Rutin, chlorogenic acid, naringenin and quercetin had binding affinities of − 12.0 kcal/mol, − 10.5 kcal/mol, − 10.4 kcal/mol and − 10.4 kcal/mol, respectively, for BChE compared to galantamine (− 10.1 kcal/mol).

**Table 4:** Binding free energies of cinnamon bark and coriander seed extracts with the cholinergic enzymes

Compound detected	Binding affinity (kcal/mol)		
	AChE	BChE	
*Donepezil	− 13.3		− 11.0
*Galantamine	− 10.8		− 10.1
Linalool	− 6.8		− 6.4
Methyl eugenol	− 7.7		− 7.0
Protocatechuic acid	− 7.1		− 7.2
Carvone	− 7.2		− 6.6
(*E*)-isoeugenol	− 7.7		− 7.3
Caffeic acid	− 8.4		− 7.9
Syringic acid	− 7.7		− 7.4
Ferulic acid	− 6.8		− 8.1
*p*-coumaric acid	− 7.5		− 7.7
**(*E*)**-cinnamic acid	− 7.3		− 6.9
Rutin	− **11.7**		− **12.0**
Vanillic acid	− 7.1		− 7.2
Chlorogenic acid	− **12.4**		− **10.5**
Eucalyptol	− 7.1		− 6.1
*p*-hydroxybenzaldehyde	− 6.2		− 5.9
2,3-dihydroxybenzoic acid	− 7.2		− 7.0
Gallic acid	− 7.4		− 7.5
Quercetin	− 10.4		− 10.4
Cinnamaldehyde	− 7.0		− 6.5
Scopoletin	− 8.2		− 8.1
Nerolidol	− 9.3		− 7.4
Naringenin	− **10.9**		− **10.4**
(*Z*)-2-methoxycinnamaldehyde	− 8.1		− 7.5
4-hydroxy-2-methoxycinnamaldehyde	− 7.8		− 7.6
Coumarin	− 7.4		− 7.1

Values in bold represent the docking scores of 3 compounds with the highest binding affinities with the protein target in each column

^*^ Standard cholinesterase inhibitors

**Fig. 5:**
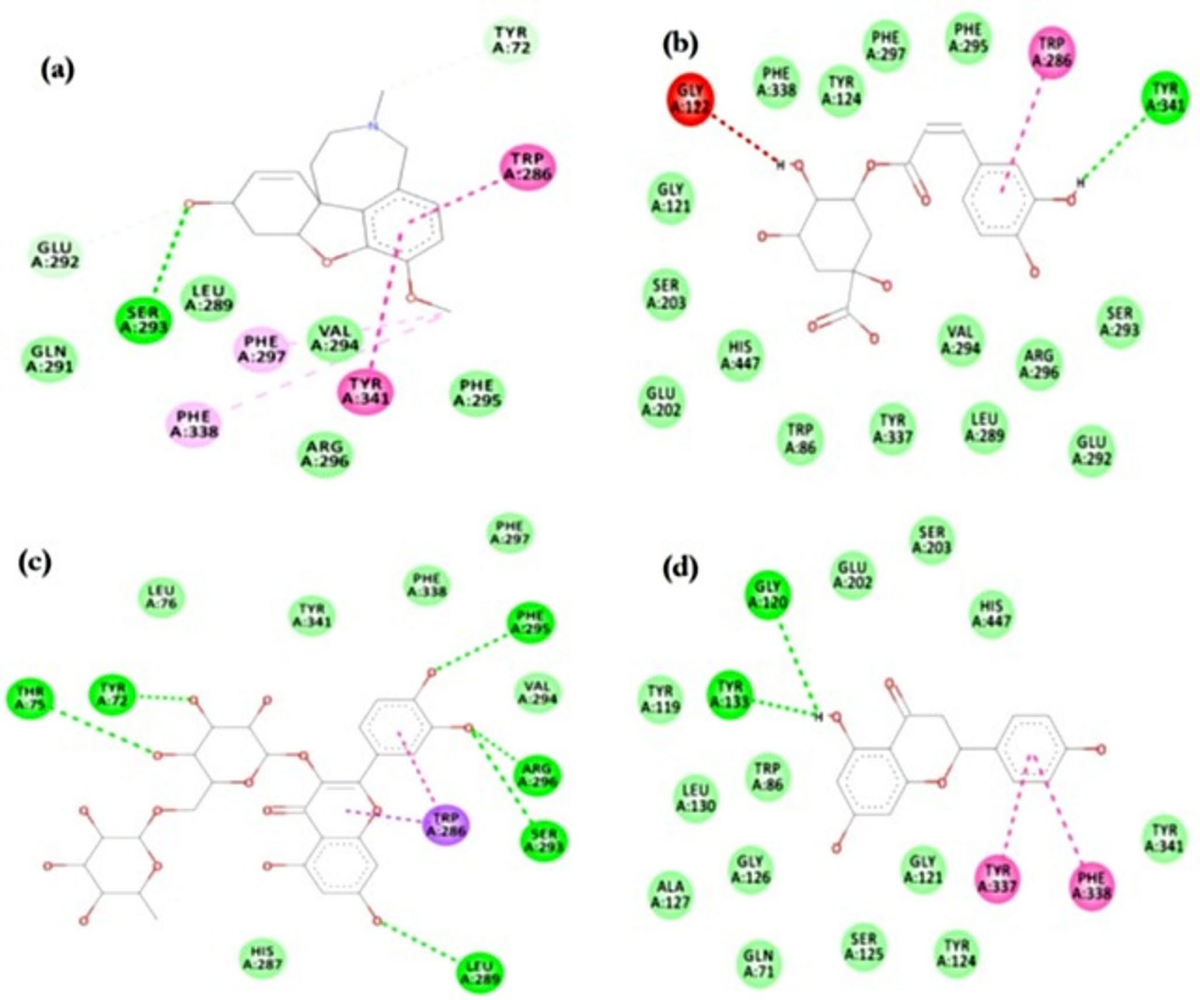
2D view of cinnamon bark and coriander seed extracts **a** galantamine, **b** chlorogenic acid, **c** rutin and **d** naringenin molecular interaction with acetylcholinesterase active site amino acids

Images in Fig. 5 show the 2D molecular interactions of chlorogenic acid, galantamine, rutin and naringenin at the AChE active site regions. In Fig. 5a, galantamine formed a hydrogen bond with GLU202, while it had hydrophobic interactions with TRP86, TYR337, and PHE338. Chlorogenic acid had a single hydrophobic interaction (π-sigma) with TRP286 and a hydrogen bond with TYR341 of AChE (Fig. 5b). The mode of interaction between rutin and AChE was solely hydrogen bond, involving PRO368, GLN369, HIS405, and ASN533 (Fig. 5c). Naringenin interacted with AChE via hydrogen bond with TYR120 and TYR133 and π−π stacking with TYR337 and PHE338 (Fig. 5d).

**Fig. 6:**
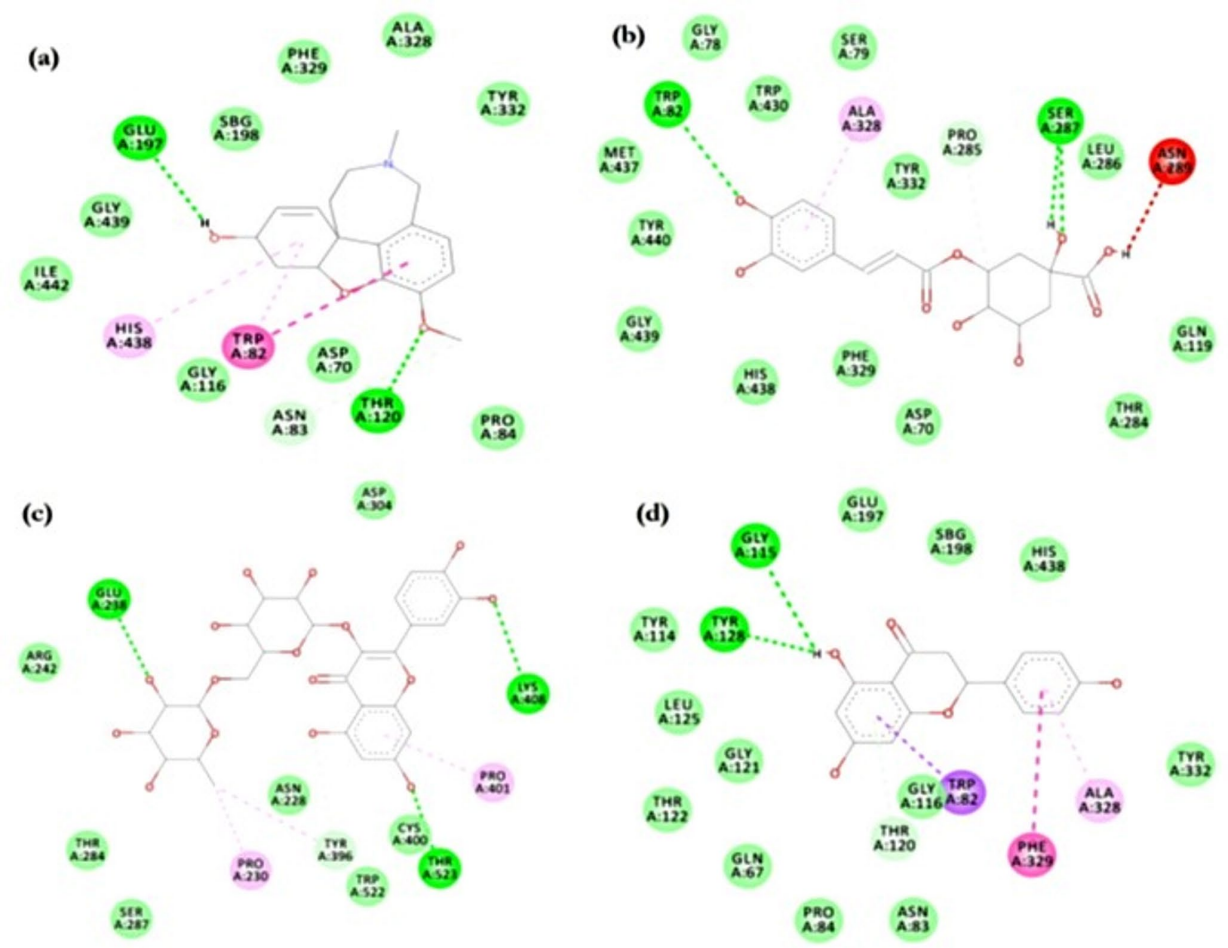
2D view of cinnamon bark and coriander seed extracts **a** galantamine, **b** chlorogenic acid, **c** rutin and **d** naringenin molecular interaction with butyrylcholinesterase active site amino acids

Similar bonding of galantamine, chlorogenic acid, rutin and naringenin with conserved active site regions of the BChE protein are presented in Fig. 6. Galantamine attached to BChE via hydrogen bond with THR120, GLU197 and hydrophobic interactions with HIS438 and conserved residue TRP82 (Fig. 6a). TRP82 of BChE was also involved in a hydrogen bond formation with chlorogenic acid while interacting with ALA328 via π-alkyl bond (Fig. 6b). A combination of π-anion interaction (with ASP70), hydrogen bond (with PRO285), π−π stacking (with TRP82), and carbon-hydrogen bond (with ASN83 and THR120) was visualized in rutin binding to BChE catalytic pocket (Fig. 6c). GLY115 and TYR128 were involved in a hydrogen bond formation with BChE, together with hydrophobic interactions with TRP82 (π-sigma), PHE329 (π-π stacking), and ALA328 (π-alkyl interaction) (Fig. 6d).

### Molecular dynamic conformational stability and fluctuations

MD simulations were carried out to investigate the molecular interactions of the potential two ligands with the BChE and AChE protein targets. To validate system stability, flexibility is essential to trace disrupted motions and avoid artefacts that may arise during the simulation runs. In this study, Root-Mean-Square Deviation (RMSD) and Root-Mean-Square Fluctuation (RMSF) were calculated to measure the systems' stability and flexibility during the 300 ns simulations. The tracing disrupted movements and prevented artefacts that could appear during the simulation, so the system’s stability needs to be validated. Therefore, we evaluated the stability of the two inhibitors inside the active sites of cholinergic enzymes. The ligand's orientation within a specific binding site may impact ligand stability, as the therapeutic impact of a small molecule depends on its stability in a target protein's binding region. The root mean square deviation measures the difference between a protein's backbone from its initial structural conformation to its final position. However, residual conformational analysis measures the nature of fluctuation exhibited by individual residues corresponding to the effect of ligand induction on the protein, which cumulatively yields its therapeutic efficacy. RMSF was calculated for each amino acid residue during a 300 ns MD simulation for all the systems. Furthermore, to indicate how the protein surface interrelates with solvent atoms and how it relates to the compactness of the hydrophobic protein core, the solvent-accessible surface area (SASA) of the protein upon ligand binding was calculated.

**Fig. 7:**
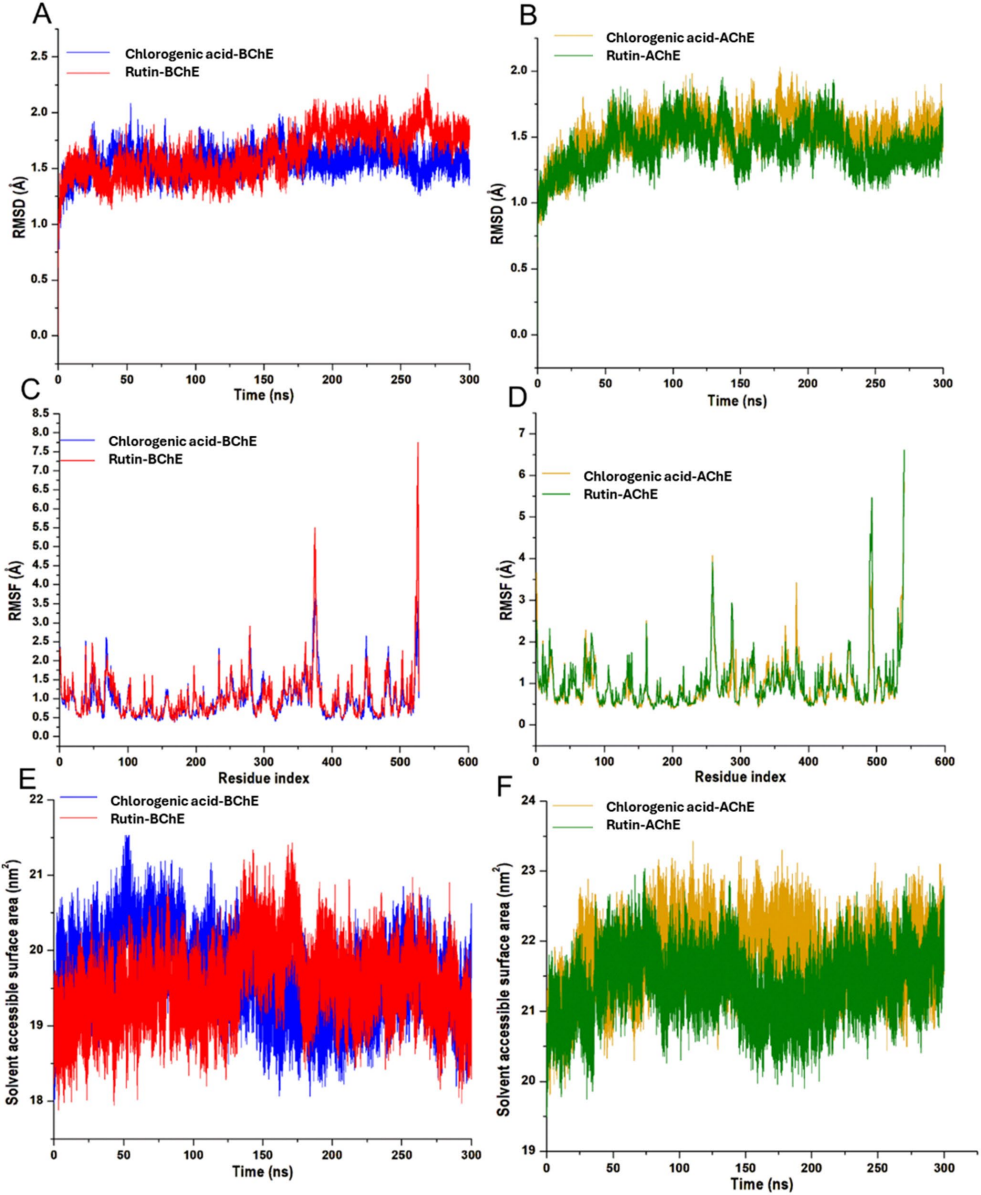
Structural representation of alterations occurring during chlorogenic acid and rutin enzymes binding to BChE and AChE ligands. The conformational stability, C-α atoms RMSD of **a** BChE and **b** AChE to the inhibitors, ran for a 300 ns MD simulation. The time evolution RMSF of each residue of **c** BChE and **d** AChE C-α atom over 300 ns solvent accessible surface area (SASA) backbone atoms relative to the starting minimized structure over 300 ns for **e** BChE and **f** AChE enzymes

This was accomplished by computing the surface area of the protein observable to solvent across the 300 ns MD simulation, which is vital for biomolecular stability. This analysis presents the average values of the compounds' RMSD and SASA within the cholinergic enzymes in Table 5. Additionally, a structural visualization using simulation RMSD, RMSF, and SASA post-analyses of the compounds inside the binding site of the proteins is shown in Fig. 7.

**Table 5:** RMSD and SASA profile of chlorogenic acid and rutin bound to the cholinergic enzymes

Systems	Estimated averages (Å)	
	RMSD	SASA
*BChE*		
Chlorogenic acid	1.56	19,640.45
Rutin	1.63	19,528.50
*AChE*		
Chlorogenic acid	1.52	21,793.89
Rutin	1.42	21,374.46

### Binding free energy landscape of chlorogenic acid and rutin to cholinergic protein target

The therapeutic effects elicited by a drug strongly rely on a protein's binding site and the activities that occur when binding ensues. To determine the inhibitory potential and molecular interactions of chlorogenic acid and rutin against the BChE and AChE enzymes, the binding free energy was evaluated by employing the Molecular Mechanics/Generalized Born Surface Area (MM/GBSA) method. From the results, it was found that chlorogenic acid and rutin bound to BChE showed less negative ∆G values, indicating weaker binding, whereas most of the corresponding ∆G values for the AChE-bound inhibitors were more negative, suggesting stronger binding (Table 6). While these more negative binding energies were obtained after 300 ns simulation time, the result may indicate analogous binding modes that possibly underlie these inhibitors' ability to bind to these receptors.

**Table 6:** MM/GBSA-based binding free energy profile of chlorogenic acid and rutin bound to cholinergic enzymes

Systems	Energy components (kcal/mol)				
	$\Delta E_{vdw}$	$\Delta E_{ele}$	$\Delta G_{gas}$	$\Delta G_{sol}$	$\Delta G_{bind}$
*BChE*					
Chlorogenic acid	− 33.74 ± 0.10	− 47.94 ± 0.17	− 81.69 ± 0.18	42.40 ± 0.09	− 39.29 ± 0.12
Rutin	− 64.79 ± 0.10	− 48.22 ± 0.31	− 113.02 ± 0.30	58.65 ± 0.18	− 54.37 ± 0.16
*AChE*					
Chlorogenic acid	− 45.51 ± 0.07	− 17.83 ± 0.42	− 63.34 ± 0.41	31.47 ± 0.21	− 31.87 ± 0.22
Rutin	− 55.94 ± 0.23	− 44.54 ± 0.26	− 100.48 ± 0.24	52.21 ± 0.14	− 48.27 ± 0.16

$\Delta E_{ele}$: electrostatic energy; $\Delta E_{vdw}$: van der Waals energy; $\Delta G_{bind}$: total binding free energy; $\Delta G_{sol}$: solvation free energy; $\Delta G_{gas}$: gas phase free energy

### Per‑residue interaction analyses

**Fig. 8:**
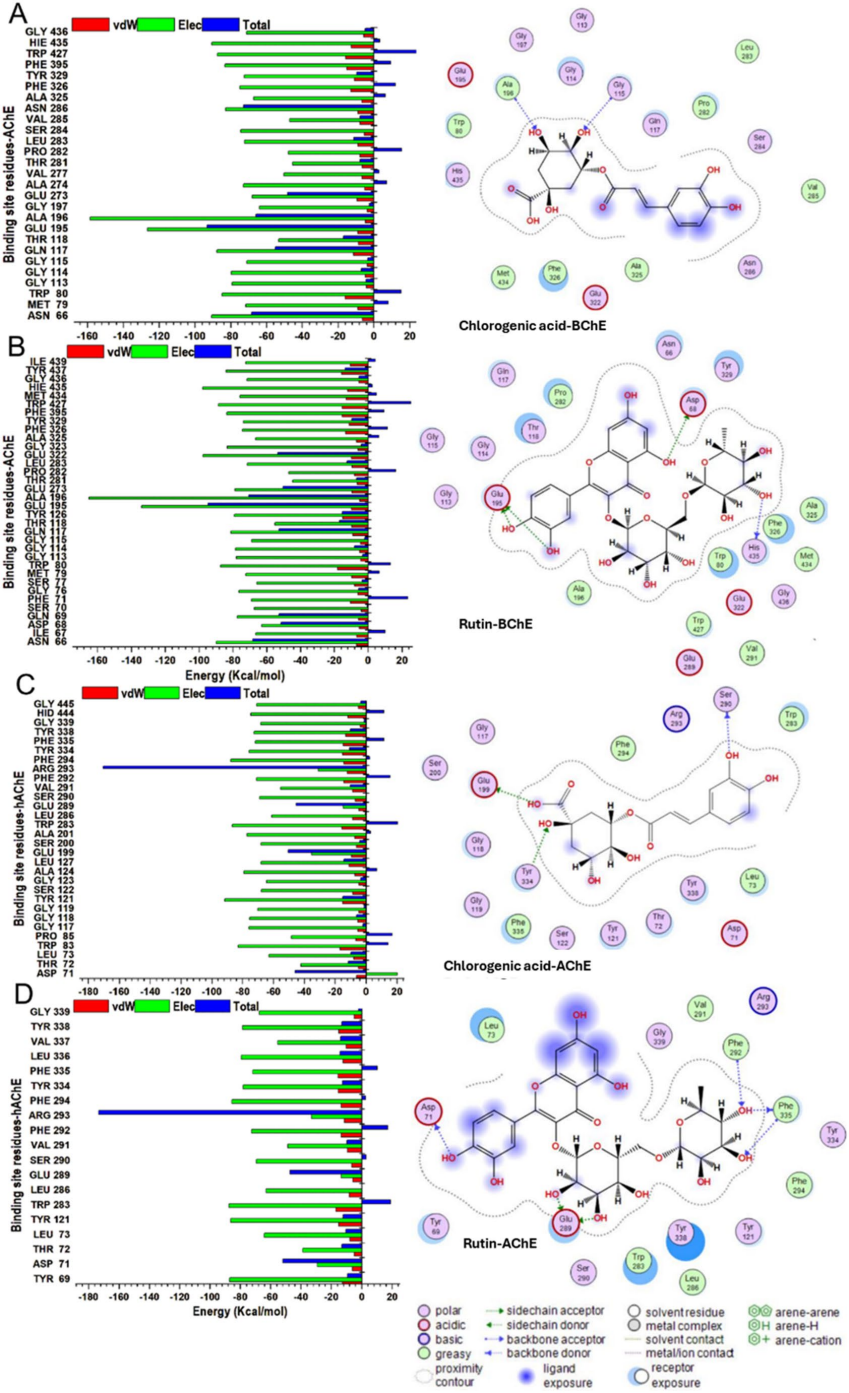
Per-residue decomposition plots showing individual energy contributions to the binding and stabilization of chlorogenic acid **a** and rutin **b** at the binding sites of BChE protein, whereas **c** and **d** show the binding and stabilization of the respective compounds at the binding sites of AChE enzyme

Decompositions of the binding free energy have been shown to present valuable insight and significant annotation of the trajectories produced by MD simulations of protein–ligand complexes. We decomposed the overall binding energy into per-residue energy contributions of individual amino acid residues in the active site to give further insight into the differences in the modes of molecular interactions of the compounds with the enzymes (Fig. 8). Thus, this data suggests that energy contributions were highest in the binding site residues.

## Discussion

Neurological disorders, including Alzheimer's disease, are considered severe health challenges globally, with increasing prevalence among older people worldwide (Kaur et al. 2017; Mollica et al. 2018). Given the harmful effects of neurological diseases on neuro-signalling and the concomitant adverse effects on memory and cognition, the search for new therapeutic agents to ameliorate the suffering of patients has become imperative. Inhibition of key enzymes implicated in neurological disorders, including AChE, BChE, beta-secretase and monoamine oxidase, has been identified as a promising approach towards developing neurotherapeutic agents (Murray et al. 2013). Therefore, we investigated ex vivo and in silico cinnamon bark and coriander seeds aqueous extracts' modulatory effects on isolated rats’ brain BChE and AChE enzymes*.* Our results in Fig. 1 and Table 1 show that both extracts exhibited higher inhibitory activities against BChE than AChE. Though with lower potency than the donepezil employed as the experimental standard, the extracts showed almost complete inhibition of BChE at some of the test concentrations. While Kaufmann et al. (2016) reported 4.33 µg/mL as the IC_50_ for AChE inhibition by galantamine, Ali Reza et al. (2018) reported 17.01 µg/mL and 19.64 µg/mL as IC_50_ values for the inhibition of AChE and BChE by donepezil and galantamine, respectively. This result agrees with Boğa et al. (2011), who reported remarkable inhibition of cholinergic enzymes by ethanol extract from the cinnamon plant, with about 63% and 85% inhibition against AChE and BChE, respectively. Similarly, methanol extracts from *Cinnamomum zeylanicum* L. leaves and cinnamon oil exhibited impressive cholinesterase inhibitory activities, with cinnamon oil displaying a better repressive activity against AChE (IC_50_: 45.88 µg/mL) and BChE (87.39 µg/mL) (Dalai et al. 2014). Likewise, methanolic extracts from eleven Jordanian spices, including white pepper, black pepper, cardamom, cinnamon, cumin, dill, fennel, lemon grass, lemon verbena, sumac and turmeric, exhibited appreciable AChE and BChE inhibitory activities (Abuhamdah 2018). However, aqueous extracts from only three studied spices: cumin, dill and fennel inhibited the target cholinergic enzymes with greater than 60% inhibition (Abuhamdah 2018). On the contrary, aqueous extract from the bark of *Cinnamomum zeylanicum* could not inhibit AChE and BChE (Abuhamdah 2018). The unique abilities of the investigated extracts to repress the catalytic activities of the cholinergic enzymes could probably be one of the “mechanisms of action” that underlie the neuroprotective effects of the spices and their abilities to improve cognitive damage (Ademosun and Oboh 2012). Thus, these findings suggest that cinnamon and coriander are natural sources of lead compounds with promising therapeutic efficacy in managing neurological disorders.

Redox imbalance between pro-oxidant and antioxidant homeostasis has been implicated in the onset of neurological disorders (Steinert and Amal 2023). This process is characterized by a high generation of reactive oxygen species (ROS) and free radicals interacting with lipid and protein components of the cell membrane, thus inducing lipid peroxidation and cell damage. For instance, highly reactive hydroxyl radicals and high levels of Fe^2+^ are generated during the development of some neurological disorders (Olofinsan et al. 2022). Exploring exogenous sources of antioxidants with promising health benefits is necessary to circumvent the adverse effects of ROS and free radicals. Hence, we assessed the in vitro antioxidant properties of aqueous extracts from cinnamon powder and coriander seeds by investigating the abilities of their extracts to scavenge ABTS and OH radicals as well as reduce Fe^3+^ and chelate Fe^2+^. Our findings showed that the extracts had remarkable antioxidant activities. The free radical scavenging abilities displayed by the extracts in this study suggest their potency in preventing oxidative stress-related diseases, including neurological disorders (Adedayo et al. 2021; Sadeghnia et al. 2013). Moreover, the neuronal cell damage induced by free radicals from Fe^2+^ generated in Fenton’s reaction during the development of AD and other neurological disorders can be avoided with subsequent improvement in neuronal health (Adedayo et al. 2021; Valko et al. 2007). Therefore, the antioxidative activities of the extracts may represent another mechanism that may underpin the neuroprotective potentials of these species since oxidative stress is one of the essential mechanisms in the aetiology of many neurological disorders (Adedayo et al. 2021). Nevertheless, the deleterious effects of oxidative stress on the brain can be reduced by dietary antioxidants, consequently improving the healthiness of the brain (Atta-ur-Rahman and Choudhary, 2001). Interestingly, our work is in line with that of Abeysekera et al. (2019), where the increasing antioxidant activity of *C. zeylanicum* Blume leaves with increasing maturity of the plant was attributed to the remarkable ability of its extracts to scavenge oxidative radicals generated in the 1,1-diphenyl-2-picryl-hydrazyl and ABTS in vitro antioxidant assays.

The biological activities reported with medicinal plant materials have been linked to the various metabolites such as sterols, polyphenols, tannins, alkaloids and terpenes inherent in them. In previous studies, polyphenols, which represent one of these compounds, have been described as having many health-promoting effects (Alegbe et al. 2019). Pharmacologically, polyphenol properties, according to studies, have been linked with the presence of one or more hydroxyl functional groups in their chemical structure. Consequently, the presence of various phenolic secondary metabolites characterized in the cinnamon and the coriander extracts (Table 3) may account for the antioxidant and cholinesterase inhibition properties exhibited by the plant products in this study. This assertion aligns with previous research, which showed some plant polyphenols' neuroprotective capacity as deduced from their excellent anti-cholinesterase properties (Ademosun et al. 2016). Overall, the extracts' bioactivity may be attributed to these synergetic effects of the OH functional groups in the structural moiety of the phenolic compounds (Odubanjo et al. 2018).

Over the last decade, computer-aided simulations have been adopted as a valuable tool for screening libraries of small molecules with potential druggable properties against proteins linked with various disease conditions (Olawale et al. 2022; Olofinsan et al. 2023). Molecular docking is an in silico analysis method that helps determine lead druggable chemical molecules by estimating their free binding energy after interaction with a target protein active site. Consequently, the lower the docking binding free energy score of the tested compound-protein complex, the stronger the affinity between the two molecules. Our molecular docking analysis results in Table 4 revealed chlorogenic acid, rutin and naringenin as lead phytoconstituents from the plant products with potent AChE and BChE inhibitory potentials. Hydroxyl groups can function as hydrogen bond donors or acceptors in chemical reactions. Therefore, the interaction of these compounds' hydroxyl groups with another functional group of amino acids in the cholinergic enzymes’ active sites could explain the observed inhibitory effect of the cinnamon powder and coriander seeds extract metabolites. Interestingly, some of these compounds from previous in vitro experiments have been reported to display anticholinergic effects (Amin et al. 2020).

Under typical body physiological conditions, proteins are flexible macromolecules. They can change their structural conformation in response to the binding of another chemical entity to their key amino acid regions. In this regard, molecular dynamics simulation is another computer algorithm-based program employed to determine alterations in the structural stability of a protein molecule after it has formed a ligand–protein complex with another chemical molecule. This in silico method allows the visualization, mimicking and prediction of the kinetics and thermodynamics of drug-protein interactions in a biological system. As the molecular dynamic data of chlorogenic and rutin complexes with BChE and AChE revealed insignificant effects on the cholinergic proteins’ architecture even at 300 ns, our findings support that the spice plant materials, which contain the polyphenols together with other constituents, could indeed modulate the protein neurological activities.

## Conclusion

Aqueous extracts from *Coriandrum sativum* seeds and *Cinnamomum verum* bark significantly inhibited BChE and AChE activities while exhibiting antioxidant properties in vitro. However, the cinnamon bark extract demonstrated better anti-cholinesterase and antioxidative activities than coriander seeds. The remarkable bioactivity of cinnamon bark extract may be attributable to the higher total phenol content and a wide range of phenolic compounds detected. Moreover, the in vitro biological activities of cinnamon bark and coriander seed extracts were validated by an in silico study, where chlorogenic acid and rutin (common to both samples) as well as naringenin (detected in cinnamon alone), were identified as the most potent biological relevant constituents in the plant extracts. Moreover, the molecular dynamics analysis revealed the good stability of the cholinergic enzymes after interaction with potentially druggable chemicals. Consequently, cholinesterase inhibition and antioxidative activities could be suggested as likely mechanisms that underlie the neuroactive potentials of these spice plants. Though these outcomes are not enough to assert that these extracts will display these neuroprotective efficacies in vivo, they create opportunities that could be explored further in other research works. As the spices may constitute an excellent source of nutraceuticals for managing AD and other neurodegenerative conditions, further studies, including bioactivity-guided isolation experiments, are required to determine if these studied plant materials represent viable sources of these neuroprotective chemical compounds.

## Data Availability

Data for this work will be made available on reasonably request.
